# EZH2 promotes hepatocellular carcinoma progression through modulating miR-22/galectin-9 axis

**DOI:** 10.1186/s13046-017-0670-6

**Published:** 2018-01-09

**Authors:** Shaofei Chen, Jiarui Pu, Jie Bai, Yuping Yin, Ke Wu, Jiliang Wang, Xiaoming Shuai, Jinbo Gao, Kaixiong Tao, Guobin Wang, Hang Li

**Affiliations:** 1 Department of Gastrointestinal Surgery, Union Hospital, Tongji Medical College, Huazhong University of Science and Technology, Wuhan, 430022 China; 2 Department of Pediatric Surgery, Union Hospital, Tongji Medical College, Huazhong University of Science and Technology, Wuhan, 430022 China

**Keywords:** Galectin-9, EZH2, Hepatocellular carcinoma, Proliferation, Metastasis

## Abstract

**Background:**

Recent studies have shown that interferon-γ (IFN-γ)-induced galectin-9 expression in Kupffer cells plays an essential role in modulatingthe microenvironment of hepatitis-associated hepatocellular carcinoma (HCC). However, whether or not IFN-γ induces galectin-9 expression in HCC cells, its biological role and regulatory mechanism in HCC development and progression are poorly defined.

**Methods:**

Quantitative PCR and western blotting analysis were used to detect galectin-9 and EZH2 levels in HCC cell lines stimulated with IFN-γ. Bioinformatics analysis and luciferase reporter assay were utilized to confirmthe binding ofmiR-22 to the 3′ untranslated region (3’-UTR) of galectin-9. The methylation status of miR-22 promoter was analyzed by MSP (Methylation specific PCR) and BSP (bisulfite sequencing PCR), while chromatin immunoprecipitation (ChIP) assay identify the occupation status of EZH2 and H3K27me3 at the promoter. Furthermore, the effect of ectopic expression of galectin-9 and miR-22 on cell proliferation, migration, invasion and cell apoptosis was assessed by using CCK-8, transwell assays and flow cytometric analysis, respectively.

**Results:**

IFN-γ induces up-regulation of galectin-9 and EZH2 in HCC cell lines. Galectin-9 is a target of miR-22 and EZH2 facilitates galectin-9 expression by tri-methylation of H3K27 on miR-22 promoter but not hyper-methylation status of DNA. MiR-22 overexpression suppressed HCC cell growth, invasion, and metastasis both in vitro and in vivo. Interestingly, galectin-9 also exhibited antitumor effects, and restoring galectin-9 expression in miR-22 overexpressing cells strengthened its antitumor effects.

**Conclusions:**

These findings indicated that EZH2 facilitates galectin-9 expression by epigenetically repressing miR-22 and that galectin-9, which is known as an immunosuppressant, also functions as a tumor suppressor in HCC.

**Electronic supplementary material:**

The online version of this article (10.1186/s13046-017-0670-6) contains supplementary material, which is available to authorized users.

## Background

Hepatocellular carcinoma (HCC) is one of the most common neoplasms worldwide. In China, about 466,100 cases are newly diagnosed and causes about 422,100 deaths in 2015 [1]. One of the well-defined risk factors for HCC is cirrhosis, whose incidence varies among different geographical regions. Hepatitis B virus infection is the most common cause of cirrhosisin Asia and Africa, while hepatitis C virus and alcohol abuse are the most common causes of cirrhosis in western countries and Japan [2]. Although great advances have been madewith respect to HCC treatment, the prognosis of the disease remains dismal [3]. Thus, studies enablingresearchers to gain a better understanding of the mechanisms underlying HCC tumorigenesis and aggressiveness are needed to improve the therapeutic responsiveness and prognosis of the disease.

Galectin-9 is a member of the β-galactoside binding protein family and featuresa tandem repeat carbohydrate recognition domain (CRD) [4]. Galectin-9 exerts immunosuppressive effects through its receptor, T cell immunoglobulin domain and mucin domain 3(Tim-3). The Tim3/Galectin-9 signaling pathway mediates T-cell senescence in HBV-associated HCC [5–7], while CD8^+^ and CD4^+^T cells exposed to exogenous galectin-9undergo apoptosis mediated by the calcium-calpain-caspase-1 pathway [8]. In addition to exerting immunosuppressive effects, galectin-9 also inducesHCC cell apoptosisin the absence of Tim-3 [9]. Therefore, we speculate that the effects of galectin-9 in malignancies may be regulated by environmental conditions and may also be cell specific. A previous study showed that tumor-infiltrating T-cell-derived IFN-γcontributes to increases in galectin-9 expression in Kupffer cells in the HCC microenvironment. However, whether IFN-γ has similar effects in HCC cells and the mechanisms through which galectin-9 expression is regulated remain largely unknown. It’s reported that galectin-9 is post-transcriptionally regulated by miR-22 in HCC cell lines and interferon-miRNA axis drives liver precancerous lesion formation and hepatocarcinogenesis [10, 11]. It’s interesting to study if IFN-γ could regulate galectin-9 expression through miRNA.

Enhancer of zeste homolog 2 (EZH2) is a member of the polycomb group (PcG) protein family, which modifies transcription at the epigenetic level by regulating histone and DNA methylation [12–14]. An extensivenumber of studies have shown that many tumor suppressor genes, including miRNAs are suppressed by EZH2 in malignancies andthat EZH2 dysregulation plays a crucial role in carcinogenesis [15, 16].

In this study, we demonstrated that IFN-γ significantly induced galectin-9 expression in HCC cells, a change accompanied by EZH2 up-regulation, and that EZH2 indirectly regulated galectin-9 expression by epigenetically repressing miR-22, a regulator of galectin-9 in HCC. Moreover, we showed that galectin-9 overexpression functions as a tumor suppressor and thus inhibits tumorigenesis both in vitro and in vivo.

## Methods

### Patients and specimens

Fresh well-established primary HCC specimens were obtained from Union Hospital, Tongji Medical College, and paired adjacent liver tissues at least 3 cm away from the tumor border were isolated for use. Each tumor tissue sample was snap-frozen in lipid nitrogen and then stored at −80°Cfor 10 min. Approval to conduct this study was granted by the Institutional Review Board of Tongji Medical College.

### Cell culture

HepG2, Hep3B and PLC/PRF5cells were obtained from the Chinese Academy of Sciences Cell Bank. The cells were cultured in Dulbecco’s modified Eagle medium supplemented with 10% fetal bovine serum (Life Technologies, Inc., Gaithersburg, MD), penicillin (100 U/ml) and streptomycin (100 mg/ml) and were maintained in a humidified incubator at 37 °C with 5% CO2.

### Western blot analysisand qPCR

Western blot and qPCR were performed as previously described [6]. The primers utilized for qPCR are presented in Additional file 1.

### Immunofluorescence

HepG2 and Hep3B cells were treated with 10 ng/ml IFN-γ for 24 h, after which they were subjected to immunofluorescence analysis to measuregalectin-9 and EZH2 expression (magnification ×400). Cells treated with PBS were used as controls. The nuclei were stained with DAPI (blue), galectin-9 was stained with FITC (green), and EZH2 was stained with cy3 (red). Pictures were taken by a fluorescence microscope.

### Plasmid construction and luciferase reporter assay

A 578-bp fragment of Gal-9 3’UTR, and the mutant form were cloned into the psiCHECK2 vector (Promega, Madison, WI, USA). A series of 5′-dissections of MIR22HG promoter was cloned into the pGL3-basic vector (Promega, Madison, WI, USA) via the MluI and XhoI sites. For the miRNAluciferase reporter assay, cells were co-transfected with 50nMmiRNA mimics or negative controls and 500 ng/ul psiCHECK2-Gal-9-3’-UTR-WT or psiCHECK2-Gal-9-Mut plasmids. For MIR22HG promoter dissection analysis, the cells were co-transfected with plasmids containing different 5′-end dissections and the pRL-TK vector (Promega, Madison, WI, USA), which served as an internal control. The cells were collected 24 h after transfection and analyzed according to the instructions of the dual-luciferase assay manual (Promega, CA, USA). Primers used are listed in Additional file 1.

### ChIP assay

HCC cells were seeded in 15 cm-wells until they reached approximately 80% confluence. ChIP assay was then performed using a SimpleChIP®Enzymatic Chromatin IP Kit (Cell Signaling Technology, Beverly, MA, USA), according to the manufacturer’s instructions. QPCR was performed with SYBR Green PCR Master Mix and primer sets targeting the putativeEZH2 binding region in the MIR22HG promoter (see Additional file 1). The amount of immunoprecipitated DNA was calculated and then normalized to the amount of input DNA.

### Cell proliferation and apoptosis assay

The effects of miR-22 and Gal-9 overexpression on cell proliferation in vitro were determined by Cell Counting Kit-8 (CCK-8, Dojindo Molecular Technologies, Kumamoto, Japan). Five wells per group were used in all experiments, whichwere repeated at least three times. FACS analysis for apoptosis was done using Annexin V apoptosis detection kits (BD Pharmingen, San Diego, CA, USA) after 48-h transfection according to the manufacturer’s protocol.

### Cell migration and invasion assay

Migration and invasion assay were conducted as previously described [17].

### Animal studies

The animal experiments were approved by theAnimal Care Committee of Tongji Medical College (IACUC: 602). Female athymic BALB/c nude mice (aged 4–5 weeks) were used for these experiments. A total of 1 × 10^6^ HepG2 cells were subcutaneously injected into the left flanks of the mice (*n* = 5 per group). Four weeks thereafter, the mice were sacrificed, the tumorswere excised, and the tumor weights and volumes were measured. In the experimental metastasis study, 1 × 10^5^ cells were injected into the tail veins of 4-week-old athymic nude mice. The lungs were obtained after four weeks and fixed with 4% paraformaldehyde before being cut into 5-μm sections, which were stained with hematoxylin and eosin. The numbers of metastatic nodules were subsequently counted to determine the effects of the indicated treatments on HCC cell metastasis.

### Statistical analysis

Data were expressed as the mean ± standard deviation (SD) of at least 3 replicated experiments. Student’s t-test was performed to analyzethe differences between two groups, and Pearson’s coefficient correlation was usedto analyze the relationships between the expression levels ofspecific genes. Fisher’s exact probability analysis was usedto compare the percentages of Ki-67 positivity among the groups. A *P* value <0.05 indicated that a particular difference was statistically significant.

## Results

### IFN-γ induces galectin-9 expression in HCC cells

**Fig. 1:**
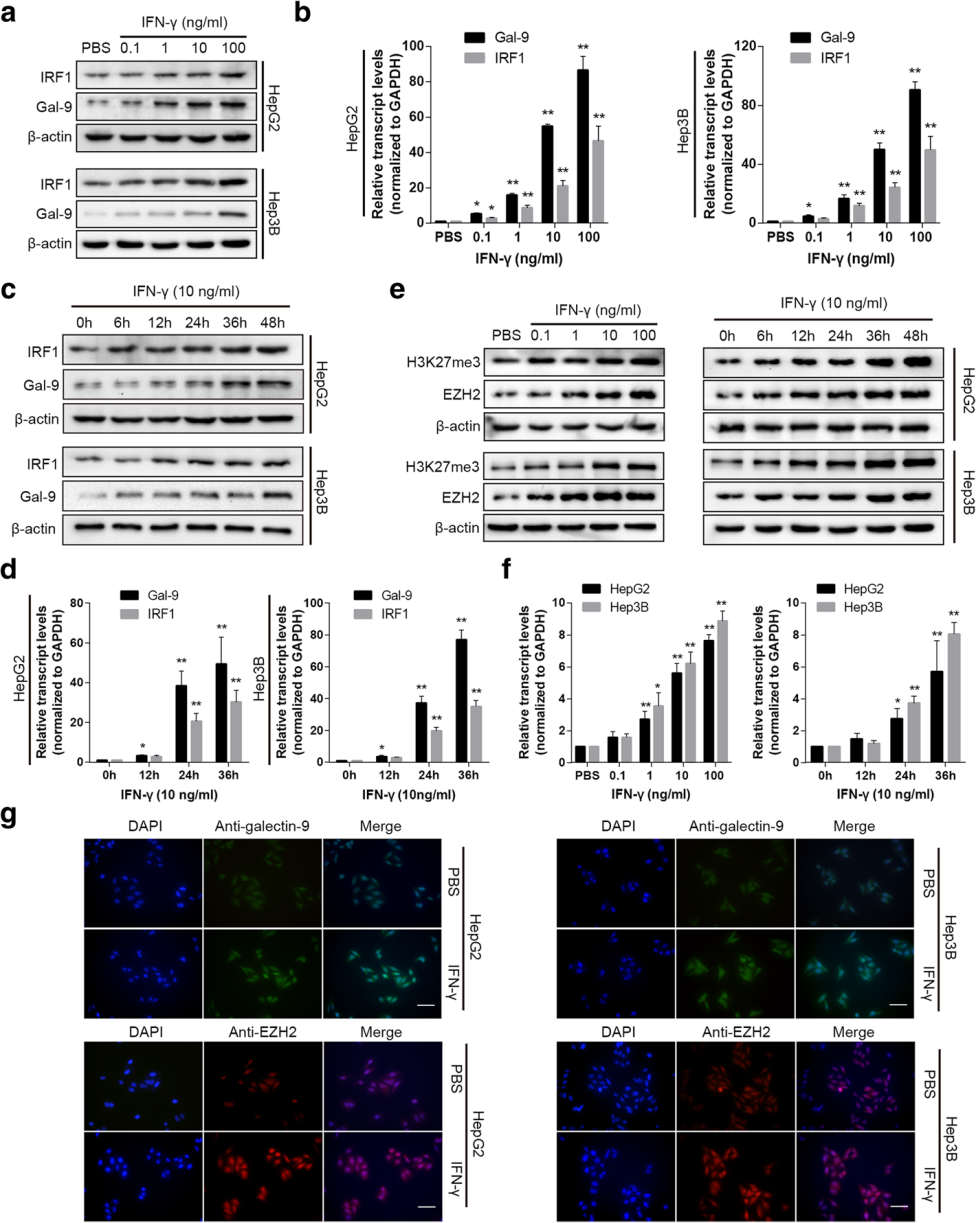
IFN-γ induces galectin-9 expression at the mRNA and protein levels in HCC cells. **a** and **b**, HepG2 and Hep3B cells were treated with IFN-γ for 24 h at different concentrations, as indicated, and then galectin-9 and IRF1 protein expression levels (**a**) and mRNA expression (**b**) levels were analyzed by western blotting and qPCR, respectively. c and d, HepG2 and Hep3B cells were treated with 10 ng/ml IFN-γ for the indicated time period, and then galectin-9, IRF1 protein (**c**) and mRNA (**d**) expression levels were determined by western blotting and qPCR, respectively. e and f,HepG2 and Hep3B cells were treated with IFN-γ for different concentration and increasing time as indicated, EZH2 and H3K27me3 protein levels (**e**) and EZH2 mRNA levels (**f**) were detected by western blotting and qPCR, respectively. (**g**) HepG2 and Hep3B cells were treated with 10 ng/ml IFN-γ for 48 h, after which galectin-9 and EZH2 were subjected to immunofluorescence staining (magnification ×400; scale bar, 50 um). Cells treated with PBS were used as controls. The nuclei were stained with DAPI (blue), galectin-9 was stained with FITC (green), and EZH2 was stained with cy3 (red). (* *P* < 0.05, **P < 0.01, compared to PBS or 0 h group)

To assessgalectin-9 expression in HCC under interferon (IFN)-γstimulation, we exposed two HCC cell lines, namely, the HepG2 and Hep3B cell lines, to recombinant human IFN-γ to mimic the microenvironment of hepatitis virus-associated HCC, an environment in which high concentrations of IFN-γ have been detected. We found that galectin-9 expression was significantly up-regulated in a concentration-dependent manner at both the mRNA and the protein level after treating with IFN-γ for 24 h, and IRF1 (Interferon regulatory factor 1) was used as a positive control (Fig. 1a and b). We then stimulated the cells with 10 ng/ml IFN-γ for increasing periods of time. As expected, we noted thatgalectin-9 mRNA and protein expression levels increased in a time-dependent manner (Fig. 1c and d). We performed an immunofluorescence analysisof HepG2 and Hep3B cells exposed to10 ng/ml IFN-γ for 48 h. Cells treated with PBS served as controls. We noted that the fluorescence intensity of galectin-9 in IFN-γ-treated cells was significantly higher than that in controlcells (Fig. 1g).

### EZH2 expression is induced by IFN-γ and is correlated with galectin-9 expression

**Fig. 2:**
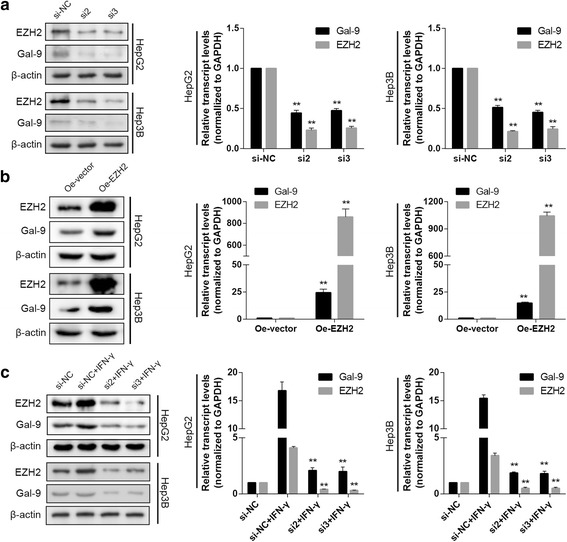
EZH2 expression was positively correlated with galctin-9 expression in HCC cells. **a** Knocking down EZH2 decreased basal galectin-9 expression at the protein and mRNA levels in HepG2 and Hep3B cells. **b** EZH2 overexpression increased galectin-9 expression at the protein and mRNA levels in HepG2 and Hep3B cells. **c** Knocking down EZH2 abolished IFN-γ-induced galectin-9 expression in HepG2 and Hep3B cells. (* *P* < 0.05, **P < 0.01, compared to control or vector group)

Because IFN-γ can modify histone 3 lysine 27 tri-methylation (H3K27me3) at gene promoters in certain cell types to regulate gene expression, we aimed to determine whether EZH2 is involved in IFN-γ-induced galectin-9 expression in HCC. To test this hypothesis, we assessed whether IFN-γcan regulate EZH2 activity. Interestingly, we detected time- and concentration-dependent increases in EZH2 and H3K27me3 levels in HCC cells stimulated by IFN-γ, increases that coincided with increases in galectin-9expression (Fig. 1e and f). This findings were supported by the results of our immunofluorescence analysis of EZH2 expression (Fig. 1g). To determine whether EZH2 affects galectin-9 expression, we performed EZH2 knockdown and overexpression experiments. Western blotting and quantitative PCR (qPCR) demonstrated that transfection of small interfering RNA specific for EZH2 significantly decreased galectin-9 protein and mRNA expressionlevels in HepG2 and Hep3B cells compared to transfection of control siRNA (si-NC) (Fig. 2a). However, transfecting EZH2-overexpression plasmidsinto HepG2 and Hep3B cells significantly increased galectin-9protein and mRNA expression levels in the corresponding cells compared to empty control (mock)-transfected cells (Fig. 2b). Furthermore, knocking down EZH2 partially blockedIFN-γ-induced galectin-9 up-regulation in the corresponding cells compared to mock-transfected cells (Fig. 2c). Taken together, these results indicated that IFN-γ-induced galectin-9 expression was positively correlated with EZH2 expression in HCC cells.

### MiR-22posttranscriptionally represses galectin-9 expression

**Fig. 3:**
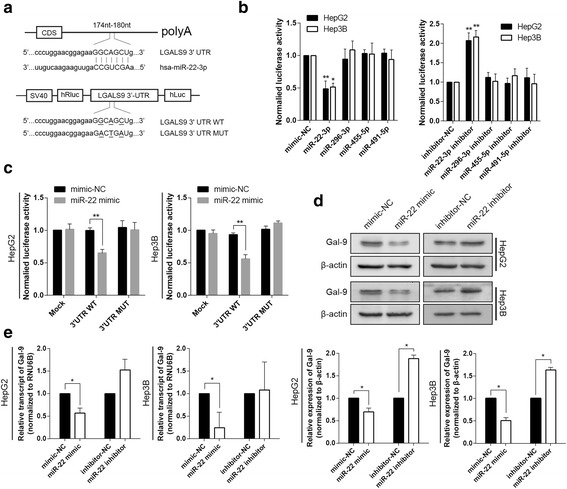
miR-22 post-transcriptionally down-regulated galectin-9 expression by directly targeting its 3’-UTR. **a** Schematic representation of the predicted binding sites of miR-22 within the 3’-UTR of galectin-9. The lower scheme represents the intact sequence of the wild-type binding site and that of its mutant counterpart within the dual-luciferase reporter vector. **b** Dual-luciferase assay showing galectin-9 3’-UTR luciferase reporter activity in cultured HepG2 and Hep3B cells transfected with mimic controls (mimic-NCs), inhibitor controls (inhibitor-NCs) and mimics (50 nmol/L) or inhibitors (50 nmol/L) of miR-22-3p, miR-296-3p, miR-455-5p, and miR-491-5p. **c** Transfection of miR-22 mimics into HepG2 and Hep3B cells resulted in decreased galectin-9 3’-UTR reporter luciferase activity compared to transfection of mimic-NCs. These effects were abolished by a mutation in the putative miR-22-binding site within the galectin-9 3’UTR. **d** and **e**, miR-22 negatively regulated galectin-9 expression at the mRNA and protein levels. Endogenous galectin-9 levels in HepG2 and Hep3B cells transfected with miR-22 mimics or NCs or mir-22 inhibitors or NCs were analyzed by qPCR and western blotting at 24 h after transfection (* *P* < 0.05, **P < 0.01, compared to mimic-NC or inhibitor-NC)

EZH2 normally silences gene expression by tri-methylating histone H3 on lysine 27 (H3K27me3); however, in our study, we found that EZH2 expression was positively correlated with galectin-9 expression. Therefore, we hypothesized that EZH2 may indirectly regulate galectin-9 expression through other intermediary molecules. As interferon–microRNA signalling drives liver precancerous lesion formation and hepatocarcinogenesis and it’s reported that galectin-9 is regulated by microRNAs, we wondered if microRNA can function as an intermediary molecule. We used computational algorithms and databases, including TargetScan [18], miRanda [19], and miRDB [20] to predict which miRNAs target galecin-9 and used miWalk to intersect the predicted results [21]. We identified four potential miRNAs—namely, miR-22-3p, miR-296-3p, miR-455-5p, and miR-491-5p—that may regulate galectin-9 by binding to its 3’-UTR. Dual-luciferase assays were performed after transfection with psiCHECK-2 vectors containing the 3’UTR sequence of galectin-9 together with microRNA mimics or inhibitors, it’s showed that transfecting HepG2 and Hep3B cells with mimics and inhibitors of miR-22-3p but not mimics and inhibitors of the other miRNAs altered luciferase activity in the indicated cell lines (Fig. 3b). These effectswere abolished by mutating the putative miR-22 binding site within the galectin-9 3’-UTR (Fig. 3c). To investigate the direct effects of miR-22 on galectin-9 expression in HCC cells, we transfected miR-22 mimics and inhibitors into HepG2 and Hep3B cells and then examined galectin-9 mRNA and protein expression levels. We found that galectin-9 protein and mRNA expression levels were decreased in cells transfected with miR-22 mimics compared to cells transfected with mimic controls. Conversely, we noted that galectin-9protein expression levels were increased in cells transfected with miR-22 inhibitors compared to cells transfected with inhibitor controls (Fig. 3d and e).

### EZH2 facilitates galectin-9 expression by repressing miR-22

**Fig. 4:**
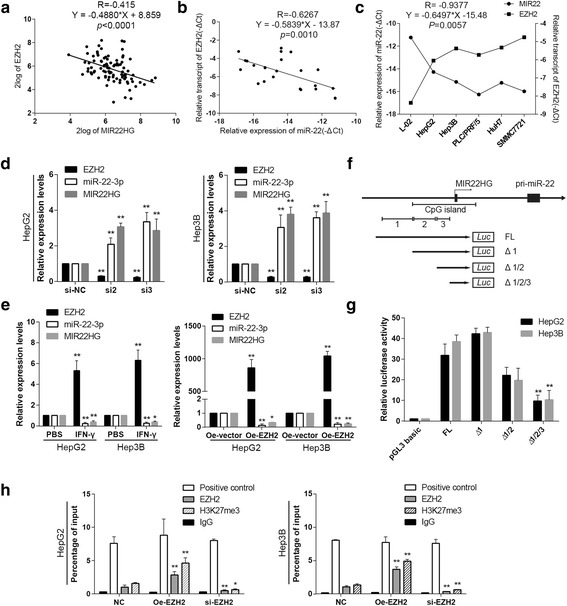
EZH2 expression was inversely correlated with miR-22 expression, and EZH2 regulated galectin-9 via miR-22. **a** EZH2 expression was inversely correlated with MIR22HG expression in clinical HCC specimens (*n* = 498) derived from the R2: microarray analysis and visualization platform (http://r2.amc.nl). **b** qPCR showed that miR-22-5p expression levels were inversely correlated with EZH2 transcript levels in HCC tissues (*n* = 24). **c** qPCR revealed that miR-22 expression was inversely correlated with EZH2 expression in HCC cells. **d** miR-22 and MIR22HG levels were measured after EZH2 knockdown and overexpression and IFN-γ treatment in HepG2 and Hep3B cells. **e** miR-22 and MIR22HG levels were measured after EZH2 overexpression and IFN-γ treatment in HepG2 and Hep3B cells. **f** Schematic representation of the MIR22HG promoter region. **g** The miR-22 promoter was dissected, as shown schematically, and the luciferase activitylevels of different constructs were determined in HCC cell lines. **h** HCC cells were stably transfected with EZH2 shRNA or pCMV-EZH2 plasmids, and ChIP assay was conducted to determine EZH2 and H3K27me3 enrichment within the MIR22HG promoter area (* *P* < 0.05, **p < 0.01, compared to mimic-NC, inhibitor-NC, or mock)

Based on the observation that miR-22 regulates galectin-9 expression, we inferred that miR-22 is the intermediate between EZH2 and galectin-9. To test this hypothesis, we determined whether EZH2 affected miR-22 expression in HCC. We mined publicly available clinical tumor expression datasets [R2: Genomics Analysis and Visualization Platform (http://r2.amc.nl)] and noted that the transcript levels of the miR-22 host gene (MIR22HG) were inversely correlated with the expression levels of EZH2 in 90 well-established HCC specimens (Fig. 4a). We then utilizedqPCR to quantify mature miR-22 and EZH2 mRNA expression levels in 24 HCC specimens and HCC cell lines anda normal hepatic cell line. We noted that EZH2 expression levels were inversely correlated with miR-22 in both the specimens and the cell lines (Fig. 4b and c). Then we conducted EZH2 knockdown and overexpression assays, which demonstrated that transfecting cells with small interfering RNA specific for EZH2 led to increases in mature miR-22 and pri-miR-22 transcript levels (Fig. 4d). In contrast, ectopically expressing EZH2 led to decreases in both miR-22 and pri-miR-22 transcript levels. IFN-γ stimulation also led to decreasesin miR-22 and pri-miR-22transcript levels (Fig. 4e). To determine whether or not EZH2 regulates galectin-9 expression through miR-22, we co-transfected cell lines that had been stably transfected with shRNA-EZH2 or pCMV-EZH2 with miR-22 mimics or miR-22 inhibitors and then measured galectin-9 protein levels. Western blotting showed that the miR-22 mimics abolished the increases in galectin-9 expression facilitated by EZH2 overexpression, while the miR-22 inhibitorsattenuated the decreases in galectin-9 expression elicited by EZH2 knockdown (see Additional files 2 and 4). Taken together, the above results indicated that EZH2 regulated galectin-9 expression by repressing miR-22 and that miR-22 plays a pivotal role in galectin-9 expression facilitated by EZH2 in HCC.

### MiR-22 transcription suppression is DNA hyper-methylation independent

Given that EZH2 represses miR-22 transcription, we sought to determinehow miR-22 transcription is regulated in HCC. Recent studies have demonstrated that miR-22 transcript levels parallel those of its host gene, MIR22HG. We retrieved the sequence of MIR22HG and determined that an 858-bp CpG island covers the promoter regionof the gene (Fig. 4f). As part of the PRC2 complex, EZH2 also recruits DNA methyltransferase (DNMT), an enzyme that participates in DNA and histone methylation, whichis involved in epigenetic regulation, to the promoter region. Therefore, we surmised that DNA methylation may also participate in miR-22 regulation. MSP (methylation specific PCR) analysis showed that no hyper-methylation was present within the CpG island upstream of the miR-22 promoter in HCC cells. Similar results were obtained in the experiments involving HCC tissues and paired adjacent normal tissues (see Additional files 3 and 4). We then performed BSP (bisulfite sequencing PCR) on HCC cells transfected with EZH2 and control. As expected, we detected hypo-methylation status in cells transfectedmock plasmids. However, we did not detect increased methylation levelsin HCC cell lines with EZH2overexpression (see Additional files 3 and 4). Based on these results, we concluded that EZH2 mediated miR-22 transcription inhibition ina DNA hyper-methylation-independent manner in HCC.

### EZH2 inhibits miR-22 transcription by promoter histone methylation

To determine how EZH2 represses miR-22 transcription, we analyzed the histone modification status of the MIR22HG promoter region. We first determined which elements of the promoter area are pivotal for miR-22 transcription regulation. A series of vectors withdifferent length of 5′ deletions of MIR22HG promoter were constructed as indicated (Fig. 4f). We notedno significant decrease in luciferase activity in cells transfected withplasmidswith a5’-deletion up to −558 comparedto cells transfected with plasmids containing the full-length promoter. Deletion of the region from −558 to −337 led to a slight decrease inluciferase activity, and deletion of the region from −337 to −65 resulted ina substantial loss of luciferase activity, indicating that this fragment was crucial for themaintenance of promoter activity (Fig. 4g).

Afteridentifyingthe region of the MIR22HG promoter responsible for maintaining its activity, we analyzed the histone modifications and EZH2 enrichment in this area by chromatin immunoprecipitation (ChIP) assay. We found that EZH2 and H3K27me3 were moderately enriched in the indicated regionin HCC cells and thatEZH2 and H3K27me3 enrichment was significantly decreased in shEZH2-transfected cells but was significantly enhanced in pCMV EZH2-transfected cells (Fig. 4h). Thus, we concluded that EZH2-mediated H3K27me3 tri-methylationcontributed to miR-22 transcriptional repression in a DNA hyper-methylation-independent manner. These results could be further confirmed by the ChIP-seq results of HepG2 cells from ENCODE (Encyclopedia of DNA Elements) project).

### MiR-22/galectin-9 suppresses HCC proliferation, migration, and invasion in vitro

**Fig. 5:**
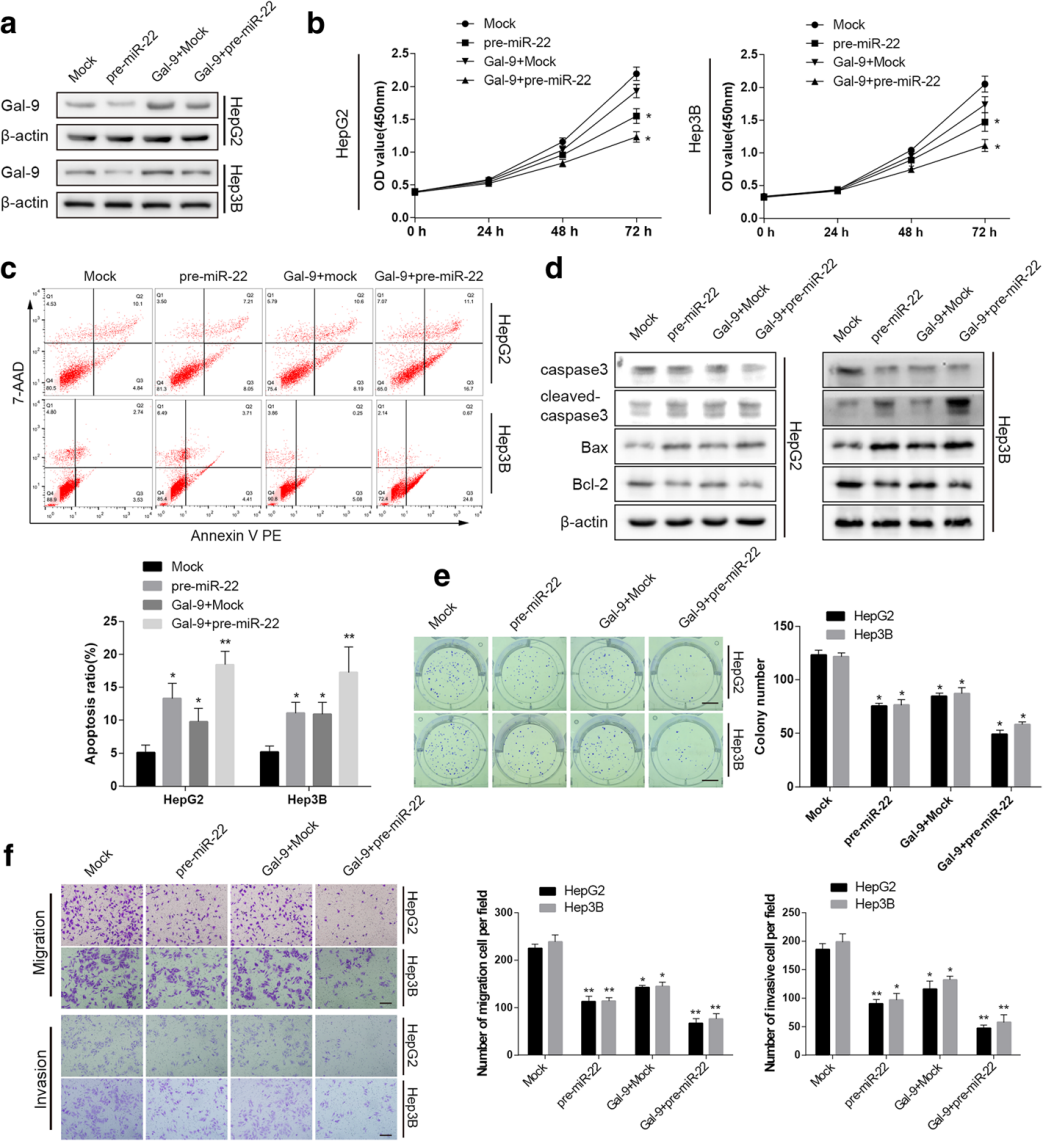
miR-22 and galectin-9 suppressed HCC cell proliferation, migration and invasion in vitro. **a** Western blot showing galectin-9 levels in cells transfected with mock or pre-miR-22 and cells co-transfected with galectin-9. **b** Growth curves of HCC cells transfected with the indicated vectors. **c** Apoptosis rates were measured by AnnexinV-PE and 7-AAD staining flow cytometry in cell with enforced expression of miR-22 and galectin-9. **d** Levels of caspase3, BCL2, BAX were detected by Western blot. **e**, Representative images and results of colony formation assay (magnification ×100; scale bar, 1 cm) of HCC cells transfected with the indicated vectors. **f** Representative images (magnification ×100; scale bar, 100um) and results of transwell migration(top) and Matrigel invasion(bottom) assays of HCC cells transfected with the indicated vectors.(* *P* < 0.05, ** *P* < 0.01,compared to mock)

We first investigated the effects of miR-22 in cultured HCC cell lines. Enforced expression of miR-22 decreased galectin-9 expression, as demonstrated by westernblotting (Fig. 5a). CCK-8 and FACS assaysshowed thatenforced miR-22 expressiondecreased cell viability and induced apoptosiscompared to stable transfectionof empty vector (Fig. 5b and c). The elevatedlevels of cleaved-caspase3 and BAX while declined BCL-2 was noted (Fig. 5d). Moreover, colony formation assay showed that miR-22 overexpression inhibited HepG2 and Hep3B cell clonogenicity (Fig. 5e). Transwell assay showed that miR-22 overexpression suppressed HCC cell migration and invasion ability (Fig. 5f). We subsequently co-transfected cells with galectin-9-overexpression vectorsandpre-miR-22 or empty vector. We found that co-transfection with pre-miR-22 and galectin-9-overexpression plasmids restored galectin-9 protein expression. Strikingly, although galectin-9 is negatively regulated by miR-22, a well identified tumor suppressor miRNA, galectin-9-overexpression and pre-miR-22 transfection suppressedHCC cell proliferation and migration. Moreover, cells transfected with both miR-22 and galectin-9 exhibited lessmalignant behaviors than cells transfected with miR-22 or galectin-9 alone (Fig. 5b–f). Collectively, these results indicated that both miR-22 and galectin-9 suppressed cell proliferation, migration and invasion in vitro.

### MiR-22 and galectin-9 suppressed HCC growth and metastasis and angiogenesis in vivo

**Fig. 6:**
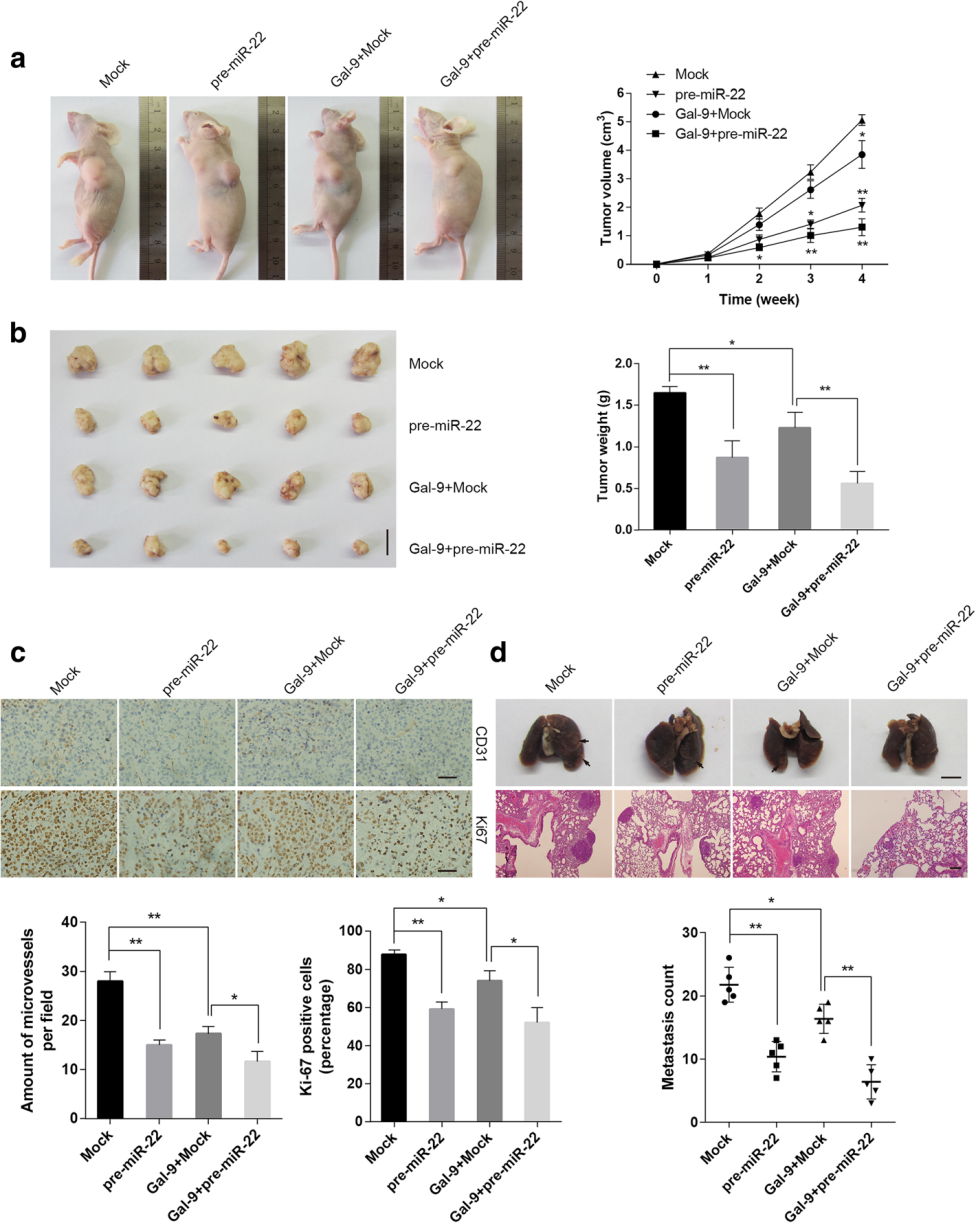
miR-22 and galectin-9 attenuated HCC cell growth and metastasis and angiogenesis in vivo. **a** Growth curves of HepG2 cells (1 × 106) stably transfected with mock vectors or miR-22-3p precursors and HepG2 cells co-transfected with galectin-9 in athymic nude mice (*n* = 5 for each group) at 4 weeks after hypodermic injection. **b** Representative images of and results for the xenograft tumors in the groups mentioned above (scale bar, 1 cm). **c** Immunohistochemical staining for CD31 and Ki-67 expression within tumors formed by hypodermic injections of HepG2 cells stably transfected with mock vectors or miR-22-3p precursors and HepG2 cells co-transfected with galectin-9 (magnification ×400; scale bar, 100 um). **d** Representative images of the whole lung (upper, arrowhead; scale bar, 0.5 cm) and HE-stained sections (lower, magnification × 100; scale bar, 100 um). Results (lower panel) of the lung metastasis analysis, which was performed after the mice were injected with HepG2 cells stably transfected with mock vectors or miR-22-3p precursors and HepG2 cells co-transfected with galectin-9 (* *P* < 0.05, ** *P* < 0.01, compared to mock)

We subsequentlyinvestigated the antitumor effects of miR-22 and galectin-9in vivo, the construction of stable cell lines are supplemented in additional file 5. We found that stable transfection of pre-miR-22 into HepG2 cells led to decreases in the sizes and weights of subcutaneous xenograft tumors in athymic nude mice compared to stable transfectionof empty vector (mock). We co-transfected galectin-9-stable cells with pre-miR-22 or empty vectors and found that these cells displayedlower tumor weights and smaller tumorsthan cells transfected with pre-miR-22 or mock alone (Fig. 6a and b). The results of experiments in which CD31 staining of micro-vessels and Ki-67 staining of proliferating cells were investigated were similarto those of the above experiments (Fig. 6c). In the metastasis study, cells stably transfected with pre-miR-22 formed significantly fewer metastatic colonies than cells transfected with mock vectors. Moreover, galectin-9-stable cells co-transfected with pre-miR-22 or mock vectorsformed fewer metastatic colonies than cells transfected with pre-miR-22 or mock vectors alone (Fig. 6d). These results suggested that both miR-22 and galectin-9 inhibited HCC cell growth and metastasis *in vivo* and that co-transfectionwithgalectin-9 enhanced the anti-tumor effects of miR-22.

## Discussion

It has been reported that IFN-γ can stimulate galectin-9 expression in endothelial cells [22–24], and our previous study showed that IFN-γ secreted by tumor infiltrating T cells induced galectin-9 expression in Kupffer cells, especially in hepatitis virus-associated HCC [6]. Our study confirmed that IFN-γ can also induce galectin-9 expression in HCC cells and that, interestingly, EZH2 expression was also up-regulated in response to IFN-γstimulation. Further study confirmed thatEZH2 can regulate galectin-9 expression by epigenetically repressing miR-22 expression.

IFN-γplays dual roles in tumor immunity [25]. It is well known that IFN-γactivates the immune system; however, uncontrolled immune system activation may cause tissue damage. We found that EZH2 and galectin-9 expression levels were up-regulated after IFN-γ stimulation in HCC, changes that we surmised are instrumental in modulating the tumor microenvironment. As the ligand for Tim3, galectin-9 participates in immune tolerance, autoimmune responses, and antiviral immune evasion [7, 26–29]. It has been reported that elevated apoptosis levels were observed in PBMCs co-cultured with HepG2 cells with galectin-9 overexpression [30]. EZH2, the catalytic subunit of PRC2, is involved in cell proliferation, cell differentiation and apoptosis [31–35], and EZH2 deregulation participates not only in tumorigenesis, tumor progression, and tumor metastasis but alsoin tumor microenvironment modulation. EZH2 was recently found to be critical for the recruitment and homing of activated Tregsto sites of inflammation [36–38]. Herein, IFN-γ-induced EZH2 and galectin-9fine-tuned the inflammatory response bysuppressing the immune system in hepatitis. However, this phenomenon may have enabled tumor cells to evade immune surveillance in HCC circumstance.

The effects of galectin-9 on cancer cells seem to be paradoxical, as some studies have reported that galectin-9 promotes cell proliferation in many blood cancers [39], while other studies have reported that galectin-9 induces cell apoptosis in cancers [40]. Several studies have shown that galectin-9 has anti-proliferative and pro-apoptotic effects in HCC that contrast with its abovementioned immunosuppressive effects in the tumor microenvironment. Immunohistochemical analysis of HCC showed that galectin-9 expression levels are lower in HCC tissues than in adjacent normal tissues [9]; however, galectin-9 expression levels were higher in HCC cells than in normal hepatocyte cells [30].

In our study, galectin-9 levels were positively correlated with those of the well-known oncogene EZH2 and were down-regulated by the tumor suppressor miR-22. However, galectin-9 overexpression exerts anti-tumor effects both in vitro and in vivo, effects that contrast with those of EZH2 but parallel with those of miR-22. This contradiction seems to be difficult to understand. It is possible that miR-22 may fine-tune but not eradicate galectin-9 expression in HCC progression. We suppose that miR-22 may regulate the balance between galectin-9 induced immune tolerance and HCC progression retardation. EZH2, as an upstream regulator of miR-22, imposes opposite effects on this delicate balance compared with miR-22. At the same time, EZH2 is a crucial epigenetic regulator of thousands of genes, as well as miRNAs, miR-22 is also regulated by other transcription factors and epigenetic modifiers. And it is more reasonable to understand the function of EZH2-miR-22-galectin-9 axis in a more profound context. It isalso believed that galectin-9 displaysdichotomousbehavior in HCC, asgalectin-9 down-regulation in hepatocytes promotes tumor growth and metastasis whereas galectin-9 overexpression in Kupffer cells and endothelial cells inhibits the anti-tumor immune responseby inducing T cell apoptosis or senescence [9, 30, 39, 40]. Some have supposed that galectin-9 has dual-functionality, and it has been proposed that galectin-9 expression may be lost during tumor progression after the initial up-regulation of the protein establishes a tumorigenic environment [9]. Therefore, the EZH2-miR-22-galectin-9 axismay regulate certain aspects of the function of galectin-9and fine-tune galectin-9 expression to enable the protein exert its effectsin HCC.

## Conclusions

In summary, we have demonstrated EZH2 regulates galectin-9 expression by epigenetically repressing miR-22 in HCC and that bothmiR-22 and galectin-9 can inhibit HCC progression. The EZH2-miR-22-galectin-9 axis is precisely regulated in HCC progression. The findings of this study suggest that miR-22 and galectin-9 may be valuable as novel targets for the treatment of human liver cancer.

## Additional files

Additional file 1: Primers. (DOCX 16 kb)

Additional file 2: Western blotting showed that miR-22 mimic transfection could abolish the increases in galectin-9 expression induced by EZH2 overexpression, while miR-22 inhibitor transfection could enhance the increases. (TIFF 1501 kb)

Additional file 3: EZH2 suppressed miR-22 transcription by DNA hyper-methylation-independent histone methylation. a, MSP analysis was conducted in HCC cell lines and tissues to determine the methylation status of the CpG island within the MIR22HG promoter. b, The methylation statuses of 28 CpG sites within the CpG island located within the core region of MIR22HG were analyzed by bisulfite sequencing. Ten clones were selected from each group, and their methylation statuses were determined. The value for the HepG2 cells was 1.07%, and the value for the HepG2 cells transfected with EZH2 was 1.79%. The value for the Hep3B cells was 1.79%, and the value for the Hep3B cells transfected with EZH2 was 2.14%. (TIFF 1755 kb)

Additional file 4: ChIP-seq data from ENCODE (encyclopedia of DNA elements) database showed enrichment peaks of EZH2 and H3K27me3 at the promoter region of MIR22HG. (TIFF 312 kb)

Additional file 5: Supplementary methods. (DOCX 14 kb)

## Abbreviations

3’-UTR: 3′ untranslated region
BSP: bisulfite sequencing PCR
ChIP: chromatin immunoprecipitation
FCM: flow cytometric analysis
Gal-9: galectin-9
HCC: Hepatocellular carcinoma
IF: immunofluorescence
IFN-γ: interferon-γ
IHC: Immunohistochemistry
IRF1: interferon regulatory factor 1
MSP: Methylation specific PCR
