# Supplementary material for: EZH2 promotes hepatocellular carcinoma progression through modulating miR-22/galectin-9 axis
Source: J Exp Clin Cancer Res. 2018 Jan 9;37:3. doi: 10.1186/s13046-017-0670-6 (PMC5761110; doi:10.1186/s13046-017-0670-6)
Supplement: Supplementary file 1 — Primers. (DOCX 16 kb) [file 13046_2017_670_MOESM1_ESM.docx]

Additional file 1

| Primers for qPCR | |  |  |  |  |  |  |
| --- | --- | --- | --- | --- | --- | --- | --- |
|  |  | Sense primer(5'-3') | | | Antisense primer(5'-3') | | |
| GAPDH |  | GTCTCCTCTGACTTCAACAGCG | | | ACCACCCTGTTGCTGTAGCCAA | | |
| galectin-9 | | TCTGGGACTATTCAAGGAGGTC | | | CCATCTTCAAACCGAGGGTTG | | |
| EZH2 |  | GACGGCTTCCCAATAACA | | | TGAGGCTTCAGCACCACT | | |
| pri-miR-22 | | CGAACAGCAGGGTGGATGAT | | | GGCAGAAAGCCTTGGGTTGT | | |
| IRF1 | | ATGCCCATCACTCGGATGC | | | CCCTGCTTTGTATCGGCCTG | | |
| Primers for reverse transcription of miRNAs(5'-3') | | | | | | | |
| miR-22-3p | | GTCGTATCCAGTGCAGGGTCCGAGGTATTCGCACTGGATACGACACAGTTC | | | | | |
| miR-296-3p | | GTCGTATCCAGTGCAGGGTCCGAGGTATTCGCACTGGATACGACGGAGAGC | | | | | |
| miR-455-3p | | GTCGTATCCAGTGCAGGGTCCGAGGTATTCGCACTGGATACGACCGATGTA | | | | | |
| miR-491-3p | | GTCGTATCCAGTGCAGGGTCCGAGGTATTCGCACTGGATACGACCCTCATG | | | | | |
| RNU6B |  | AACGCTTCACGAATTTGCGT | | |  |  |  |
| Primers for qPCR of miRNAs | | | |  |  |  |  |
|  |  | Sense primer(5'-3') | | | Antisense primer(5'-3') | | |
| miR-22-3p | | GCCTGAAGCTGCCAGTTGA | | | GTGCAGGGTCCGAGGT | |  |
| miR-296-3p | | GCCGAGGGTTGGGTGGAG | | | GTGCAGGGTCCGAGGT | |  |
| miR-455-3p | | GCCGTATGTGCCTTTGGACT | | | GTGCAGGGTCCGAGGT | |  |
| miR-491-3p | | GCCTGAGTGGGGAACCCTT | | | GTGCAGGGTCCGAGGT | |  |
| RNU6B |  | CTCGCTTCGGCAGCACA | |  | AACGCTTCACGAATTTGCGT | | |
| Primers for MSP and BSP | | |  |  |  |  |  |
|  |  | Sense primer(5'-3') | | | Antisense primer(5'-3') | | |
| MSP unmethylated | | TGGTGTTAGGTTGAGAATTATATGG | | | ACTAAAAAACCAACCAATAAACAAA | | |
| MSP methylated | | GACGGTGTTAGGTTGAGAATTATAC | | | ACTAAAAAACCGACCAATAAACG | | |
| BSP |  | GGTAAAAGGGTGGTTTTTTTGTTA | | | CCAACTCACACCCACCTAATACTA | | |
| Primers for CHIP | |  |  |  |  |  |  |
|  |  | Sense primer(5'-3') | | | Antisense primer(5'-3') | | |
| MIR22HG promoter | | ACTCTCGTTTGACGTAGCGCTT | | | CGCCCTGGCTCTGATTGGCAAG | | |

|  |  | Sense primer(5'-3') | Antisense primer(5'-3') |
| --- | --- | --- | --- |
| LGALS9 3'UTR WT | ATCTCGAGGCGGCTTCCTGGCCCT | | TGAATGTGCCAACAAGCATTTTCATT |
| LGALS9 3'UTR MUT | AACGGAGAAGACTGATGACGGGGATTGC | | CCCCGTCATCAGTCTTCTCCGTTCCAGG |
| miR-22 promoter | | | |
| FL（-1000-+100） | ATACGCGTGCTCATTGGTCTCCACCTTC | | ATCTCGAGTCGCCAGCTCACACCCAC |
| Δ1（-558-+100） | ATACGCGTCCCGCAGAGGCCGG | | ATCTCGAGTCGCCAGCTCACACCCAC |
| Δ2（-337-+100） | ATACGCGTCGCCTGCTCTTTAGGACTCTC | | ATCTCGAGTCGCCAGCTCACACCCAC |
| Δ3（-65-+100） | ATACGCGTCGCAGCTCGTGCGTCAC | | ATCTCGAGTCGCCAGCTCACACCCAC |
