# Supplementary figures and images for: EZH2 promotes hepatocellular carcinoma progression through modulating miR-22/galectin-9 axis
Source: J Exp Clin Cancer Res. 2018 Jan 9;37:3. doi: 10.1186/s13046-017-0670-6 (PMC5761110; doi:10.1186/s13046-017-0670-6)

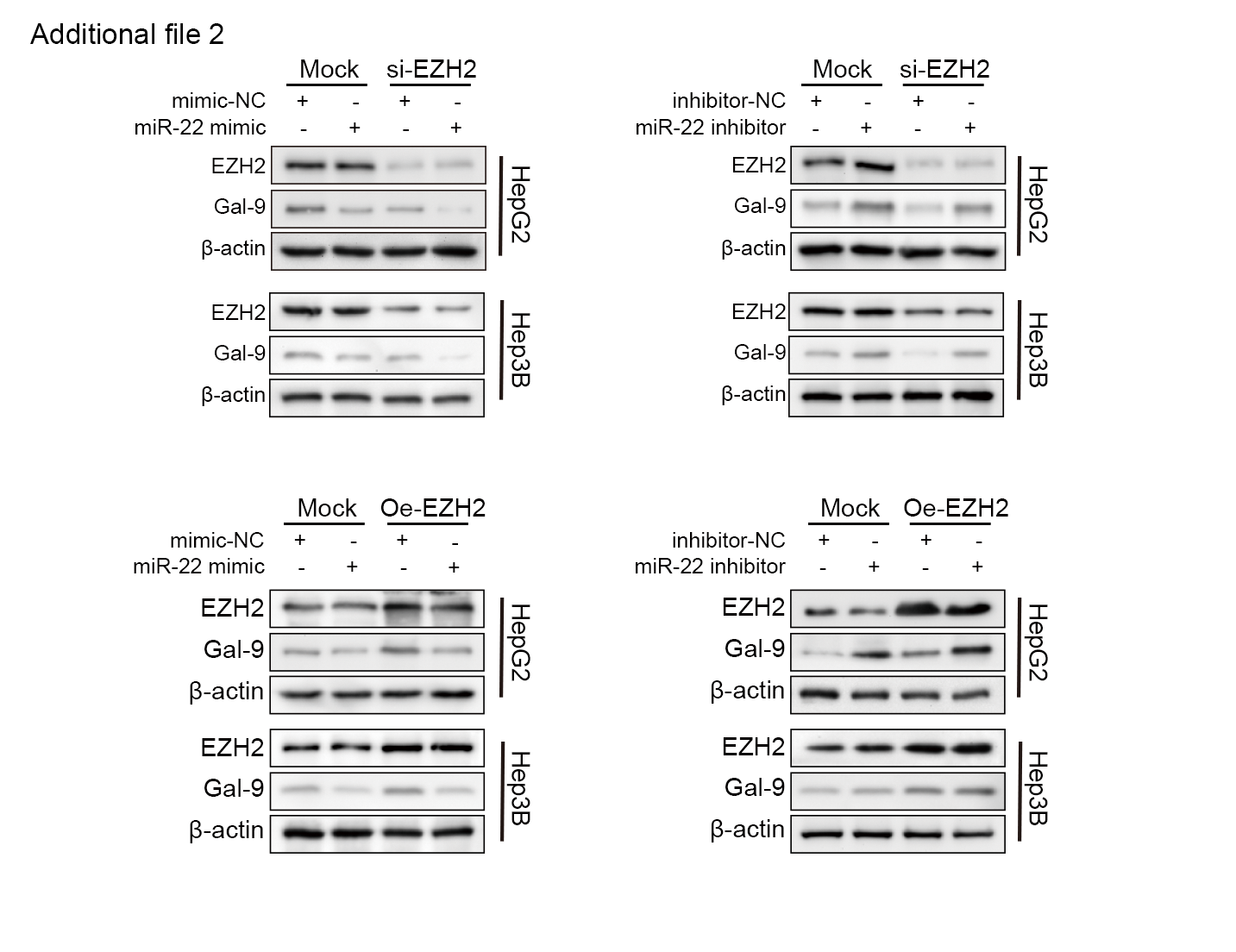

Supplement: Supplementary file 2 — Western blotting showed that miR-22 mimic transfection could abolish the increases in galectin-9 expression induced by EZH2 overexpression, while miR-22 inhibitor transfection could enhance the increases. (TIFF 1501 kb) [file 13046_2017_670_MOESM2_ESM.tif]

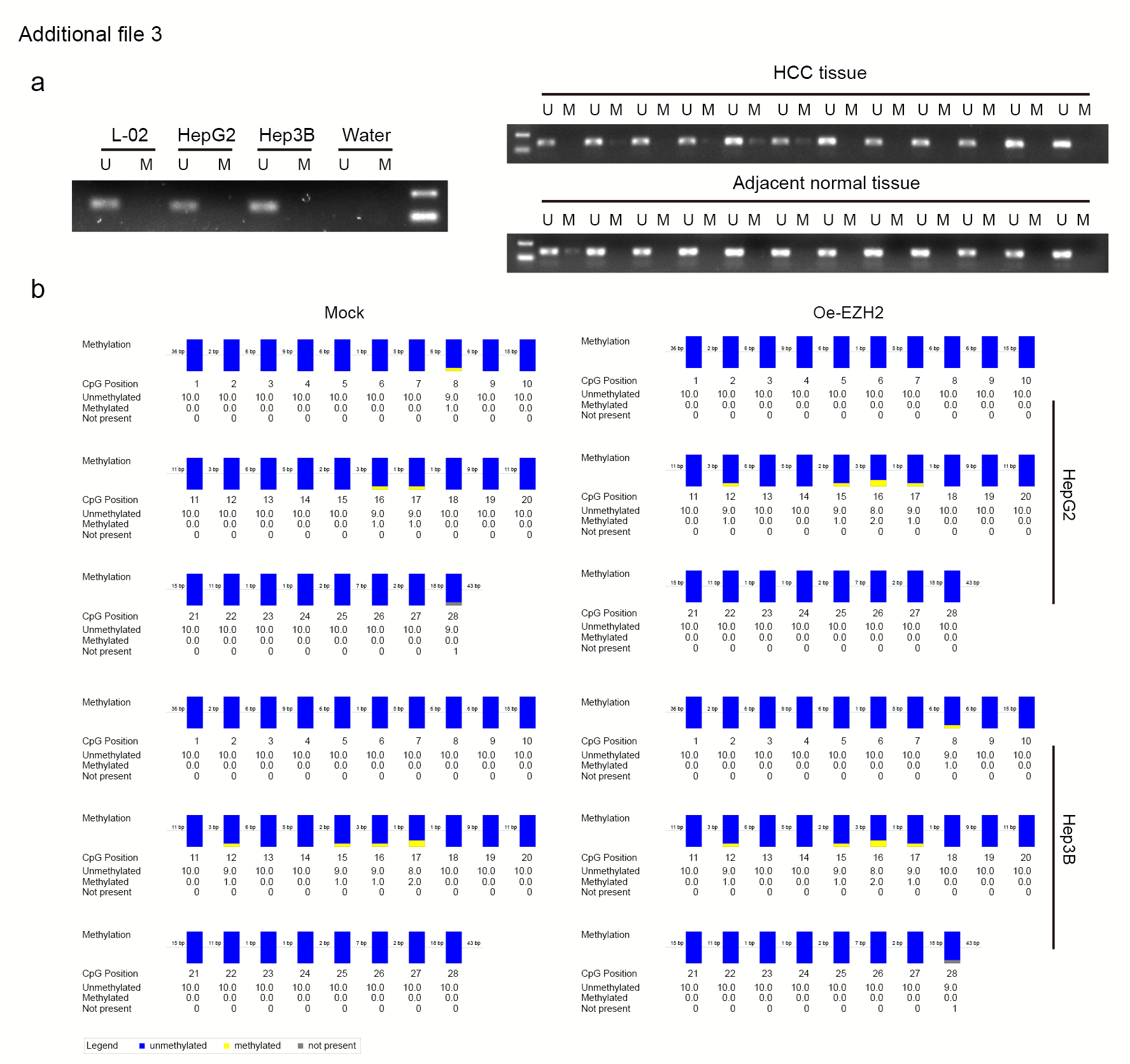

Supplement: Supplementary file 3 — EZH2 suppressed miR-22 transcription by DNA hyper-methylation-independent histone methylation. a, MSP analysis was conducted in HCC cell lines and tissues to determine the methylation status of the CpG island within the MIR22HG promoter. b, The methylation statuses of 28 CpG sites within the CpG island located within the core region of MIR22HG were analyzed by bisulfite sequencing. Ten clones were selected from each group, and their methylation statuses were determined. The value for the HepG2 cells was 1.07%, and the value for the HepG2 cells transfected with EZH2 was 1.79%. The value for the Hep3B cells was 1.79%, and the value for the Hep3B cells transfected with EZH2 was 2.14%. (TIFF 1755 kb) [file 13046_2017_670_MOESM3_ESM.tif]

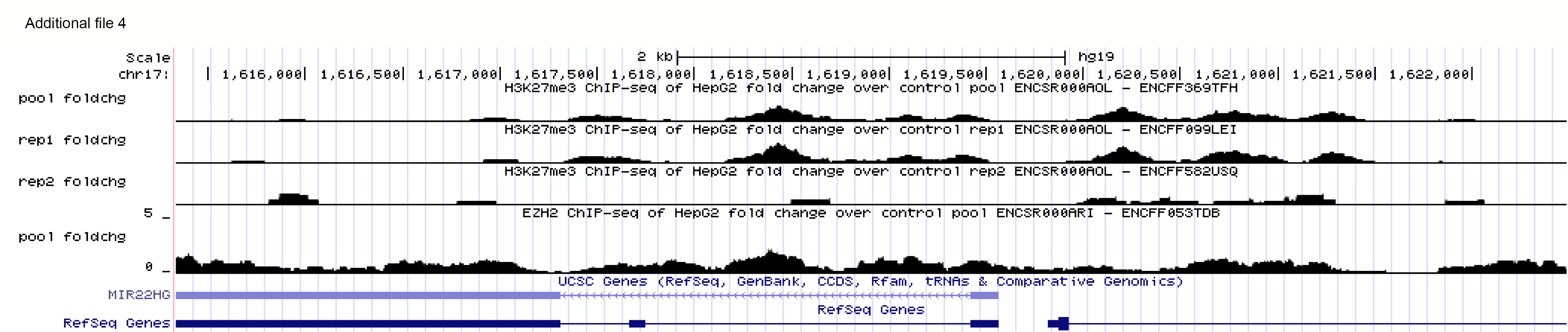

Supplement: Supplementary file 4 — ChIP-seq data from ENCODE (encyclopedia of DNA elements) database showed enrichment peaks of EZH2 and H3K27me3 at the promoter region of MIR22HG. (TIFF 312 kb) [file 13046_2017_670_MOESM4_ESM.tif]
