# Supplementary material for: EZH2 promotes hepatocellular carcinoma progression through modulating miR-22/galectin-9 axis
Source: J Exp Clin Cancer Res. 2018 Jan 9;37:3. doi: 10.1186/s13046-017-0670-6 (PMC5761110; doi:10.1186/s13046-017-0670-6)
Supplement: Supplementary file 5 — Supplementary methods. (DOCX 14 kb) [file 13046_2017_670_MOESM5_ESM.docx]

**Additional file 6**

**Suplementary methods**

**1. Antibodies and reagents**

EZH2, H3K27me3 and β-actin antibodies were purchased from Cell Signaling Technology (Cell Signaling Technology, Beverly, MA, USA), and galectin-9 antibodies were purchased from R&D (R&D Systems Inc. Minneapolis, MN, USA). HRP-conjugated secondary antibodies and Ki-67 and CD31 antibodies were purchased from Proteintech (Proteintech, Wuhan, China). IFN-γ was obtained from PeproTech (PeproTech, Rocky Hill, NJ, USA), and Matrigel matrix was obtained from BD Biosciences (BD Biosciences, Sparks, MD, USA).

**2. Construction of stable cell lines**

To obtain cell lines that stably expressed pre-miR-22 and Gal-9, we transfected HepG2 and Hep3B cells with pMIR-preMIR-22-GFP and pENTER-Gal-9 (Vigenebio, Jinan, China). Cells stably expressing the indicated genes were selected with puromycin and neomycin. The EZH2-interfering plasmid GV248/GFP+Puro (Genechem, Shanghai, China) was transfected into the indicated cells to obtain cell lines in which EZH2 was knocked down. Stable EZH2-knockdown cells were subsequently selected by puromycin before being subcloned to obtain a uniform stable cell line.

**3. Anchorage-dependent colony formation assay**

Tumor cells were plated in 35-mm dishes at a density of 300 cells per well. All cell lines were plated in triplicate. The cells were grown in culture for 14 days, after which they were washed twice with PBS, fixed in methanol for 10 min, and stained with 0.1% crystal violet for 30 min. The numbers of colonies that had formed (a colony comprised more than 50 cells) were subsequently counted.

**4. Anchorage-independent colony formation assay**

Approximately 5×10^3^ cells were suspended in medium containing 0.35% low-melt agarose and then seeded in a six-well plate overlaid with 0.6% low-melt agarose that had solidified at room temperature. After incubating for 14 days, the cells were stained with 0.1% crystal violet for 30 min, and the numbers of colonies with diameters >80 μm were counted under a microscope.

**5. Methylation analysis**

Genomic DNA was isolated and was then bisulfite-modified using an EZ-DNA Methylation-Gold Kit (Zymo Research, Orange, CA, USA). For BSP, the PCR products were cloned using a TA cloning kit (Invitrogen, San Diego, CA, USA). Plasmid DNA was isolated from 10 positive clones using a QIAquick Plasmid Mini Prep Kit (Qiagen, Valencia, CA, USA) and then sequenced. MSP analysis of the bisulfite-treated DNA was subsequently performed, and then the PCR products were visualized after being electrophoresed on a 1.5% agarose gel. The primers used for BSP and MSP are listed in supplementary table S1.
